# Role of Prehypertension in the Development of Coronary Atherosclerosis in Japan

**DOI:** 10.2188/jea.14.57

**Published:** 2005-03-18

**Authors:** Masakazu Washio, Shoji Tokunaga, Kouichi Yoshimasu, Hiroko Kodama, Ying Liu, Shizuka Sasazuki, Keitaro Tanaka, Suminori Kono, Masahiro Mohri, Akira Takeshita, Kikuo Arakawa, Munehito Ideishi, Takanobu Nii, Kazuyuki Shirai, Hidekazu Arai, Yoshitaka Doi, Tomoki Kawano, Osamu Nakagaki, Kazuyuki Takada, Koji Hiyamuta, Samon Koyanagi

**Affiliations:** 1 Department of Preventive Medicine, Graduate School of Medical Sciences, Kyushu University.; 2 Department of Cardiovascular Medicine, Graduate School of Medical Sciences, Kyushu University.; 3 Second Department of Internal Medicine, School of Medicine, Fukuoka University.; 4 Tokushukai Fukuoka Hospital.; 5 Saiseikai Fukuoka General Hospital.; 6 National Kyushu Medical Center Hospital.

**Keywords:** hypertension, coronary arteriosclerosis, risk, Japan

## Abstract

BACKGROUND: Hypertension is an important risk factor of coronary heart disease. A new guidelines for hypertension prevention and management in The Seventh Report of the Joint National Committee on Prevention, Detection, Evaluation, and Treatment of High Blood Pressure in the United States recommended lifestyle modification or medical treatment for subjects with prehypertension. However, whether prehypertension increases the risk of coronary atherosclerosis in the Japanese population is still unknown.

METHODS: A cross-sectional study in a clinical setting was conducted. The subjects were 705 patients (417 males and 288 females) aged 30 years and older who underwent a first-time coronary angiography for suspected or known coronary heart disease at 5 major cardiology departments in the Fukuoka metropolitan area between September 1996 and August 1997.

RESULTS: Compared to subjects with normal blood pressure, those with prehypertension had an increased risk of coronary atherosclerosis even after adjusting for other factors.

CONCLUSION: Prehypertension may be an important clinical entity which requires treatment in the Japanese population.

Coronary heart disease is the single most important disease entity in many industrialized countries,^1^ and hypertension is an important risk factor for coronary heart disease in western countries^2^^,^^3^^,^^4^ as well as in Japan.^5^ Recently, Vasan et al.^6^ reported that high normal blood pressure^7^ (i.e., systolic blood pressure of 130 to 139 mmHg or diastolic blood pressure of 85 to 89 mmHg) is associated with an increased risk of coronary heart disease. In the United States, The Seventh Report of the Joint National Committee on Prevention, Detection, Evaluation, and Treatment of High Blood Pressure provided guidelines for hypertension prevention and management.^8^ They recommended lifestyle modifications for subjects with prehypertension (i.e., systolic blood pressure of 120 to 139 mmHg or diastolic blood pressure of 80 to 89 mmHg).

The Japanese Society of Hypertension Guidelines Subcommittee for the Management of Hypertension provided guidelines for the management of hypertension for general practitioners.^9^ They recommended that blood pressure should be controlled under 140/90 mmHg among middle-aged hypertensive patients. The guidelines for treatment of hypertension in the elderly , 2002 revised version^10^ recommended that blood pressure should be controlled under 140/90 mmHg for those aged 60-69 years, under 150/90 mmHg for ages 70-79 years and under 160/90mmHg for those 80 years or over. The Tanno-Sobetsu Study^11^ demonstrated that cumulative survival rates with cardiovascular mortality decreased as systolic blood pressure increased, with a significant decline in elderly Japanese aged 60 years or over (systolic blood pressure 160-179 mmHg vs. systolic blood pressure <140 mmHg, relative risk=3.75). We have already shown that hypertension defined as either systolic blood pressure of 160 mmHg or higher, diastolic blood pressure of 95 mmHg or higher, or with administration of anti-hypertensive agents is a significant risk factor of coronary atherosclerosis among the Japanese population.^12^ The purpose of the present study was to evaluate the relation of prehypertension to the severity of coronary atherosclerosis in the Japanese population.

## METHODS

### Patients

Study subjects were patients aged 30 years and older who underwent the first-time angiographic examination at five major cardiology departments in the Fukuoka metropolitan area (i.e., 4 departments in Fukuoka City and 1 department in an adjacent city) during the period from September 1996 through August 1997. All patients were referred due to clinical signs of coronary heart disease. The following patients were excluded from the study: those with known myocardial infarction, valvular disease, or cardiomyopathy; and those who had undergone aorto-coronary bypass operation or percutaneous transluminal coronary angioplasty.

A total of 733 (85.7%) of 838 eligible patients agreed to take part in this study and answered a questionnaire about lifestyle and other factors; 38 patients refused to participate in this study, 16 were severely ill, and 51 were discharged before the interview was arranged. Among the 733 participants, 28 were excluded; 2 of these had a single coronary artery, for 2 information about the duration of chest pain was not obtained, 23 received medication for angina pectoris more than 6 months before the onset of chest pain, and 1 answered that chest pain had lasted more than 20 years. Thus, the remaining 705 patients (417 men and 288 women), who were 62.4±10.8 (mean±standard deviation) years old, participated in the present study.

### Coronary angiography

Coronary angiography was performed by the percutaneous transbrachial or transfemoral technique. The interpretation of angiograms was done by experienced cardiologists at each hospital in conformity with American Heart Association methods.^13^ The luminal diameter was measured by calipers at one hospital and was approximated at the other hospitals. The lumen diameter reduction in each 15 coronary artery segments was classified as 0% (normal), 25%, 50%, 75%, 90%, 99%, or 100% according to the maximal narrowing. Coronary artery stenosis was defined when 50% or greater luminal narrowing occurred in one or more coronary arteries. To examine agreement of the angiographic evaluation across departments, randomly chosen cinefilms for 10 patients (Gensini’s score:^14^ median 33.0, range 0-90) from one teaching department were assessed in the other 4 departments in a masked fashion; the presence or absence of coronary artery stenosis agreed completely, and the interclass correlation coefficient for Gensini’s score was 0.92.^15^

### Behavioral and clinical risk factors

After informed consent was obtained, interviews were started. Research physicians or nurses asked patients to answer questions about lifestyle factors such as smoking habits and alcohol drinking. The coronary heart disease-related symptom or abnormal electrocardiogram leading to coronary angiography was elicited, and lifestyle and other factors prior to the onset of the event were ascertained. All clinical and laboratory data as well as the medical history were obtained from medical records just before and during admission for coronary angiography.

Blood pressure was measured by hospital nurses or physicians using standard mercury sphygmomanometers on the right arm of the seated patient after more than 5 minutes of bed rest in the ward. Korotkoff phases I and V were considered to represent systolic and diastolic blood pressures, respectively. According to the new guidelines for hypertension prevention and management in The Seventh Report of the Joint National Committee on Prevention, Detection, Evaluation, and Treatment of High Blood Pressure,^8^ hypertension was defined as present if patients had systolic blood pressure of 140 mmHg or higher and/or diastolic blood pressure of 90 mmHg or higher. Normal blood pressure was defined as present if patients had systolic blood pressure of less than 120 mmHg and diastolic blood pressure of less than 80 mmHg. The remaining patients were defined as having prehypertension.

Diabetes mellitus was defined as present if patients were under dietary or drug treatment for diabetes mellitus or when they had fasting plasma glucose of 140 mg/dL or higher, or casual plasma glucose of 200 mg/dL or higher. Dyslipidemia was defined as present when patients received lipid-lowering agents, or their serum cholesterol levels were 220 mg/dL or higher, or their HDL cholesterol levels were less than 40 mg/dL.

### Statistical analysis

Logistic regression analysis was used to adjust for potential confounding factors. Age was categorized into 5-year age groups. Exposure to cigarette smoking was expressed as cigarette-years, i.e., the number of cigarettes smoked per day multiplied by years of smoking, which was categorized into 0, 1-399, and 400+. Alcohol consumption (mL/day) was calculated for current drinkers, categorized into 0, 1-49, and 50+ mL/day. Age, cigarette smoking, and alcohol use were treated as continuous variables, and indicator variables were used for hospital, blood pressure, and other factors. Odds ratios (OR) and their 95% confidence interval (CI) were obtained from the logistic regression coefficients and their standard errors. All the statistical analyses were performed using the Statistical Analysis System (SAS^®^) (SAS Institute, Inc., Cary, NC, USA). All the p-values quoted were two tailed, and values less than 0.05 were regarded as statistically significant.

## RESULTS

A total of 311 patients (44.1%) had hypertension and 246 patients (34.9%) had prehypertension while only 148 patients (21.0%) were categorized into the normal blood pressure group. Mean (± standard deviation) systolic blood pressure was 135±22 mmHg for all patients (hypertension; 155±16 mmHg, prehypertension 128±6 mmHg; normal blood pressure, 109±7 mmHg) while mean diastolic blood pressure was 76±13 mmHg for all patients (hypertension; 85±11 mmHg, prehypertension; 73±8 mmHg; normal blood pressure, 64±8 mmHg). Among the 705 patients, 288 (40.9%) were treated with antihypertensive agents. Diabetes mellitus was found in the 126 patients (17.9%) while 440 patients (62.4%) had dyslipidemia. A total of 370 patients (52.5%) were smokers, and 322 patients (45.7%) were current drinkers.

Table 1 summarizes the characteristics of patients with and without coronary artery stenosis. Fewer females had coronary artery stenosis than males while hypertension, medication for hypertension, diabetes mellitus, dyslipidemia and current smokers were more commonly seen in the patients with coronary artery stenosis than in those without it.

**Table 1.** Comparison between patients with coronary artery stenosis and those without.

	with coronary artery stenosis	without coronary artery stenosis	p-value
	n=311	n=394	
females	93 (29.9)	195 (49.5)	<0.01
65 years and older	159 (51.3)	176 (44.7)	0.09
blood pressure levels			<0.01
hypertension *	159 (51.3)	152 (38.6)	
prehypertension ^†^	104 (33.4)	142 (36.0)	
normal blood pressure ^‡^	48 (15.4)	100 (25.4)	
medication for hypertension	146 (47.0)	142 (36.0)	<0.01
diabetes mellitus	83 (26.7)	43 (10.9)	<0.01
dyslipidemia	209 (67.2)	231 (58.6)	0.02
current smokers	181 (58.2)	189 (48.0)	<0.01
current alcohol drinkers	134 (43.1)	188 (47.7)	0.22
percentages in parentheses.			

*: systolic blood pressure ≥ 140 mmHg and/or diastolic blood pressure ≥90 mmHg

^†^: neither hypertension nor normal blood pressure

^‡^: systolic blood pressure <120 mmHg and diastolic blood pressure <80 mmHg

Table 2 shows the ORs of coronary artery stenosis in relation to blood pressure categories, diabetes mellitus and dyslipidemia. Prehypertension, like hypertension, was associated with an increased risk of coronary artery stenosis even after adjusting for other risk factors. Diabetes mellitus and dyslipidemia were also risk factors of coronary artery stenosis.

**Table 2.** Odds ratios for coronary artery stenosis.

		odds ratio (95% CI) *	odds ratio (95% CI) ^†^
normal blood pressure		1.00 (reference)	1.00 (reference)
prehypertension		1.29 (1.05, 1.60)	1.30 (1.05, 1.61)
hypertension		1.39 (1.02, 1.90)	1.42 (1.03, 1.94)

diabetes mellitus	(yes/no)	1.67 (1.34, 2.08)	1.70 (1.36, 2.11)

dyslipidemia	(yes/no)	1.31 (1.10, 1.56)	1.29 (1.09, 1.54)

CI: confidence interval.

*: adjusted for age, sex, and hospital.

^†^: adjusted for age, sex, hospital, smoking, and alcohol drinking.

## DISCUSSION

Hypertension is associated with an increased risk of cardiovascular disease^2^^,^^3^^,^^4^^,^^17^^,^^18^^,^^19^ as well as stroke.^4^^,^^16^^,^^18^ Ueda et al.^19^ reported that the management of mild hypertension with a diastolic blood pressure of 90 to 104 mmHg was more effective in reducing stroke than coronary heart disease in Japan. In western countries, however, high normal blood pressure with a systolic blood pressure between 130 and 139 mmHg or a diastolic blood pressure between 85 and 89 mmHg increases the risk of coronary heart disease.^6^ Furthermore, in the Hypertension Optimal Treatment Study,^20^ the most prominent protection against cardiovascular events by lowering blood pressure was observed in patients with systolic blood pressure in the range of 130-140 mmHg and diastolic blood pressure in the range of 80-85 mmHg. Lewington et al.^21^ demonstrated that vascular mortality decreased most when blood pressure was reduced to 115 mmHg for systolic blood pressure and 75mmHg for diastolic blood pressure.

The Hisayama study^18^ demonstrated that systolic hypertension increased the risk of acute myocardial infarction in both middle-aged and elderly Japanese while mild hypertension (i.e., systolic blood pressure between 140 and 159 mmHg and diastolic blood pressure between 90 and 94 mmHg) did so only in the elderly. In the present study, however, prehypertension showed an increased OR for coronary artery stenosis even after controlling for age and other factors.

Because our study was conducted more than one decade after the Hisayama Study,^18^ the different results of the two studies may be partly explained by the fact that the prevalence of obesity has rapidly increased due to the westernization of the Japanese lifestyle, including dietary habits,^22^ which has brought on other coronary heart disease risk factors associated with insulin resistance syndrome such as hypertension, dyslipidemia, diabetes mellitus and hyperuricemia.^22^

Liu et al.^23^ demonstrated that body mass index was significantly associated with hypertension, diabetes mellitus, and hypercholesterolemia in the Japanese population. Handa et al.^24^ reported that the combination of hypertension and hypercholesterolemia appeared to be important in the development of coronary atherosclerosis. Kaplan proposed the term “deadly quartet” as a profile of a person having a very high risk of coronary heart disease with a combination of upper body obesity, hypertension, hypertriglyceridemia, and impaired glucose tolerance.^25^ The combination of prehypertension and other borderline diseases such as impaired glucose tolerance and a high normal lipid profile may increase the risk of coronary heart disease. Because a change to a westernized life-style was associated with an increased risk of coronary heart disease among Japanese immigrants,^26^ the results of our study may indicate that blood pressure control of prehypertension may be important for the prevention of coronary heart disease in the Japanese population.

There are certain limitations in the current study. First, the participants were not randomly selected from the general population. Most of the participants had risk factors of coronary heart disease other than high blood pressure such as diabetes mellitus (17.9%), dyslipidemia (62.4%) or smoking (45.7%). However, the total cholesterol level^27^^,^^28^ and the prevalence of diabetes mellitus^29^ are increasing in the Japanese population. Very recently, Nakanishi et al.^30^ reported that the accumulation of coronary heart disease risk factors was highly associated with the increased risk of hypertension among Japanese men. Our results may give us useful information to prevent the increase of coronary heart disease among the Japanese population in the future.

The second limitation is that the blood pressure might have been higher than the usual blood pressure regardless of the degree of coronary atherosclerosis because all patients might have waited with anxiety for their first-time angiographic examinations. However, the nondifferential misclassification of ‘normal blood pressure to prehypertension’ and ‘prehypertension to hypertension’ may lead to underestimation of the effect of high blood pressure on coronary atherosclerosis because the nondifferential misclassification underestimates the risk of exposure.^31^

Third, our study was a cross-sectional one.^32^ Because we collected the information about blood pressure and coronary artery stenosis at the same time, the sequence of high blood pressure and coronary artery stenosis cannot be necessarily determined in the present study.

Finally, we could not evaluate the contents of antihypertensive treatment because we did not obtain information about antihypertensive drugs such as diuretics, beta-blockers, calcium channel blockers, and angiotensin converting enzyme inhibitors.

On the other hand, our study has strengths as well. First, this cross-sectional study is one of the largest studies to evaluate the effect of prehypertension on angiographically defined coronary atherosclerosis among Japanese population. Second, the participating hospitals were five major cardiology departments in the Fukuoka metoropolitan area. Third, we confirmed that the angiographic evaluation of coronary artery stenosis agreed very well among these five cardiology departments.^15^

In summary, the present findings should be interpreted with caution because the patients in the present study were a high-risk group for coronary heart disease. However, the present study clearly showed that, compared with normal blood pressure, prehypertension was associated with an increased risk of coronary artery stenosis even after adjusting for other risk factors. Our results indicate that maintaining normal blood pressure levels with systolic pressure of less than 120 mmHg and diastolic pressure of less than 80 mmHg should be recommended to prevent coronary artery stenosis among Japanese. In the United States, the Seventh Report of the Joint National Committee on Prevention, Detection, Evaluation, and Treatment of High Blood Pressure^8^ recommended lifestyle modifications as well as medical treatment for prehypertensive subjects with high coronary heart disease risk. Prehypertension appeared to be an important clinical entity which requires medical treatment in the Japanese population as well, especially when patients have other risk factors for coronary heart disease.
